# Regional Mucosa-Associated Microbiota Determine Physiological Expression of TLR2 and TLR4 in Murine Colon

**DOI:** 10.1371/journal.pone.0013607

**Published:** 2010-10-22

**Authors:** Yunwei Wang, Suzanne Devkota, Mark W. Musch, Bana Jabri, Cathryn Nagler, Dionysios A. Antonopoulos, Alexander Chervonsky, Eugene B. Chang

**Affiliations:** 1 Department of Medicine, Knapp Center for Biomedical Discovery, University of Chicago, Chicago, Illinois, United States of America; 2 Department of Pathology, University of Chicago, Chicago, Illinois, United States of America; 3 Institute for Genomics & Systems Biology, Argonne National Laboratory, Argonne, Illinois, United States of America; University of Wisconsin-Milwaukee, United States of America

## Abstract

Many colonic mucosal genes that are highly regulated by microbial signals are differentially expressed along the rostral-caudal axis. This would suggest that differences in regional microbiota exist, particularly mucosa-associated microbes that are less likely to be transient. We therefore explored this possibility by examining the bacterial populations associated with the normal proximal and distal colonic mucosa in context of host Toll-like receptors (TLR) expression in C57BL/6J mice housed in specific pathogen-free (SPF) and germ-free (GF) environments. 16S rRNA gene-based terminal restriction fragment length polymorphism (T-RFLP) and clone library analysis revealed significant differences in the community structure and diversity of the mucosa-associated microbiota located in the distal colon compared to proximal colon and stool, the latter two clustering closely. Differential expression of colonic TLR2 and TLR4 along the proximal-distal axis was also found in SPF mice, but not in GF mice, suggesting that enteric microbes are essential in maintaining the regional expression of these TLRs. TLR2 is more highly expressed in proximal colon and decreases in a gradient to distal while TLR4 expression is highest in distal colon and a gradient of decreased expression to proximal colon is observed. After transfaunation in GF mice, both regional colonization of mucosa-associated microbes and expression of TLRs in the mouse colon were reestablished. In addition, exposure of the distal colon to cecal (proximal) microbiota induced TLR2 expression. These results demonstrate that regional colonic mucosa-associated microbiota determine the region-specific expression of TLR2 and TLR4. Conversely, region-specific host assembly rules are essential in determining the structure and function of mucosa-associated microbial populations. We believe this type of host-microbial mutualism is pivotal to the maintenance of intestinal and immune homeostasis.

## Introduction

The mammalian colon is home to a unique ecosystem of trillions of microorganisms that generally live in harmony with the host and serve important physiological functions [1]–[3]. Hundreds of microbial species are present both within the colonic lumen and closely associated with the mucosa. While both populations are believed to be important to host physiology, mucosa-associated microbiota, which intimately contact and interact with the host, are likely to be especially pivotal to functions such as immune activation, angiogenesis, epithelial growth and development, gene expression and mucus production [4]–[7]. It is well known that many types of host gene expression in the colonic mucosa are highly regulated by microbial signals, including intestinal epithelial heat shock proteins [8], RELM-beta [9], and the vitamin D receptor [10]. However, few studies have distinguished between luminal and mucosa-associated microbes of the mammalian colon or have specifically examined how mucosa-associated microbiota is related to regional host responses or gene expression. Many of these genes are differentially expressed along the rostral-caudal axis of the mammalian colon, presumably to serve unique functions inherent to that region. This would suggest that there are corresponding differences in region-specific microbiota which account for the physiological and regional expression of these genes. However, in at least two studies of the human enteric microbiome, there appeared to be very few differences between the mucosa-associated microbiota of the proximal and distal colon [11], [12], It should be noted that in both studies, healthy human subjects underwent standard colonoscopy requiring colonic lavage. In retrospect, this may have affected both luminal and mucosa-associated microbiota, skewing them to appear less diverse and more homogeneous throughout the length of the colon.

In this study, we revisit this issue to focus specifically on mucosa-associated microbiota of the proximal and distal colon of healthy wild-type mice. Samples were harvested under physiological conditions and with special care to minimize manipulation of the bowel. 16S rRNA gene-based analyses were used to assess the microbial profiles in these two regions of the colon. At the same time, we examined the region-specific expression of several Toll-like receptors (TLRs) of the colonic mucosa that play a key role in innate immunity. Parallel studies were performed in germ-free (GF) mice to better understand the role of the enteric microbiota in regulation of the regional expression of the mucosal TLRs. These studies reveal a heterogeneity of regional mucosa-associated microbiota that appears to be determined by local host conditional factors. While the enteric microbiota of these regions varied considerably among different litters of in-house and Jackson Laboratory bred mice, the regional patterns remained consistent, i.e. proximal colonic and stool samples were similar, but both were significantly different from that in the distal colon. Finally, the profile of regional mucosa-associated microbiota was the major determinant for the regional expression of at least two TLRs, TLR2 and TLR4.

## Results

### T-RFLP cluster analysis of regional colonic mucosa-associated and stool microbiota in mouse littermates

**Figure 1:**
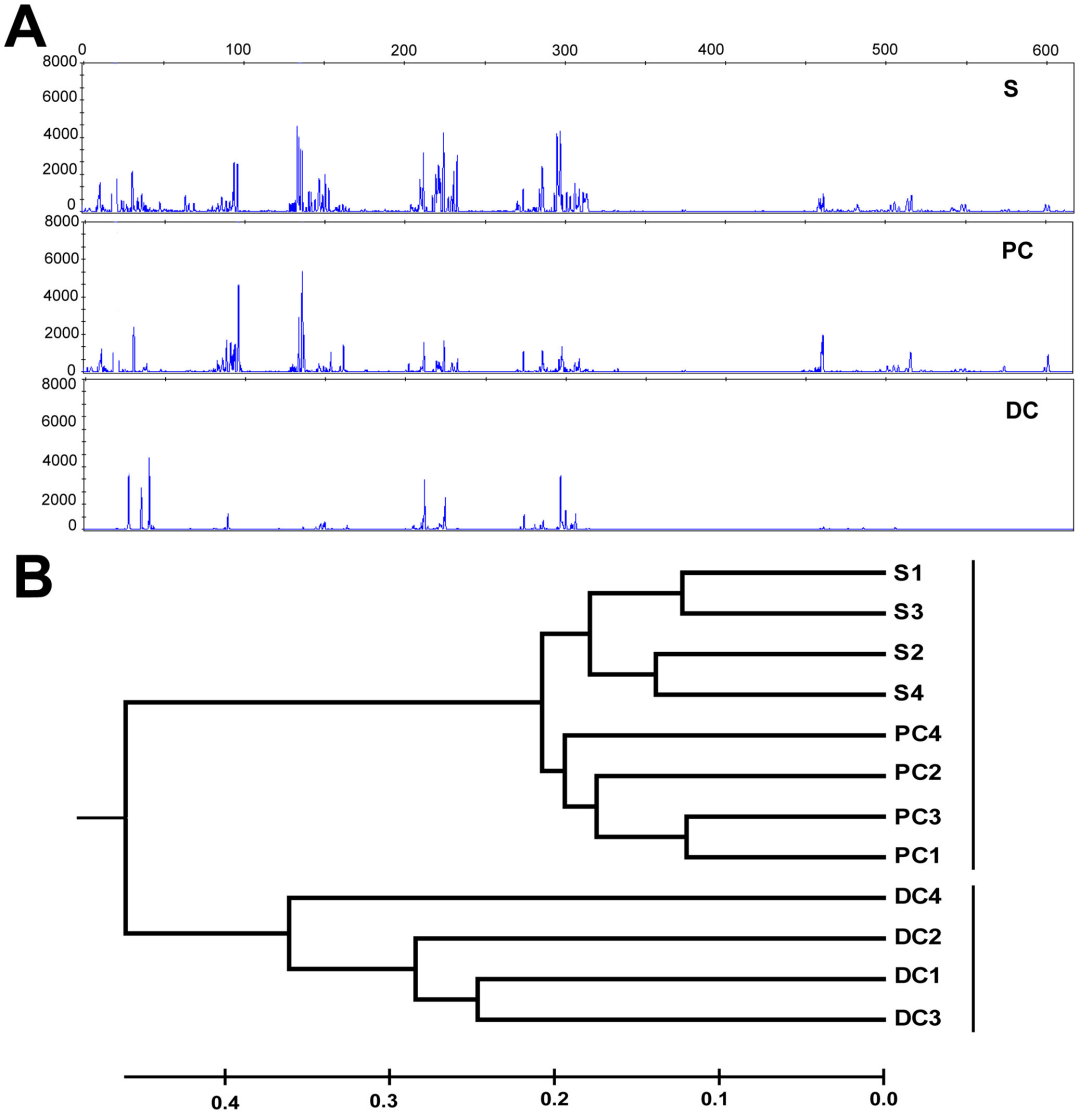
T-RFLP analysis of microbial communities in mouse colonic tissue and stool samples. (A) Representative T-RFLP patterns of bacterial populations in mouse stool sample (S), proximal (PC) and distal colon (DC). 16S rRNA genes were obtained from amplification of DNA template (50 ng DNA), digested by restriction enzyme *Msp* I and analyzed by capillary electrophoresis. Fragment size in base pairs is shown at the top and peak height is shown as relative fluorescence. (B) A representative phylogenetic tree was built up from 4 littermate mice based on T-RFLP analysis. Similarities of bacterial populations between stool sample (S), proximal colon (PC) and distal colon (DC) were compared by Bray-Curtis distance calculations. The scale bar shows the distance of similarity.

Regional mucosa-associated bacteria from mice of the same litter were first examined by T-RFLP analysis in which the 16S rRNA gene was amplified from bacterial DNA and then cut with the restriction enzymes *Msp* I yielding DNA fragments of different lengths which were characteristic for different bacteria. As judged by T-RFLP dendrograms, the microbial profiles of the distal colon (DC) were distinct from those found in the proximal colon (PC) and stool samples (S), the latter two being relatively similar (representative T-RFLP dendrograms are shown in Figure 1A).

The T-RFLP profiles were further analyzed by pairwise Bray-Curtis distances to examine the similarity between samples from mice of the same litter. As shown in Figure 1B, corresponding samples collected from littermate mice clustered based on the location of sample collection. Proximal colon and stool samples with similar T-RFLP profiles grouped together. However, at a 40% distance cutoff, mucosa-associated distal colonic bacteria clustered separately from proximal colon and stool bacterial samples. To further quantify the differences of bacterial populations between proximal and distal colon, the absolute richness (the number of distinct terminal restriction fragments in each sample) of the bacterial community was calculated. The richness of the community was greater in stool samples (40.5±8.4) and the proximal colon (38.3±8.8) than that in the distal colon (28.7±6.2). The difference was significant comparing the distal colon to the other samples (n = 24, p<0.05) in all litters, but there were no significant differences in richness between the proximal colon and stool sample.

### Taxonomical assignment for 16S rRNA gene sequences in different regions of the mouse colon

**Figure 2:**
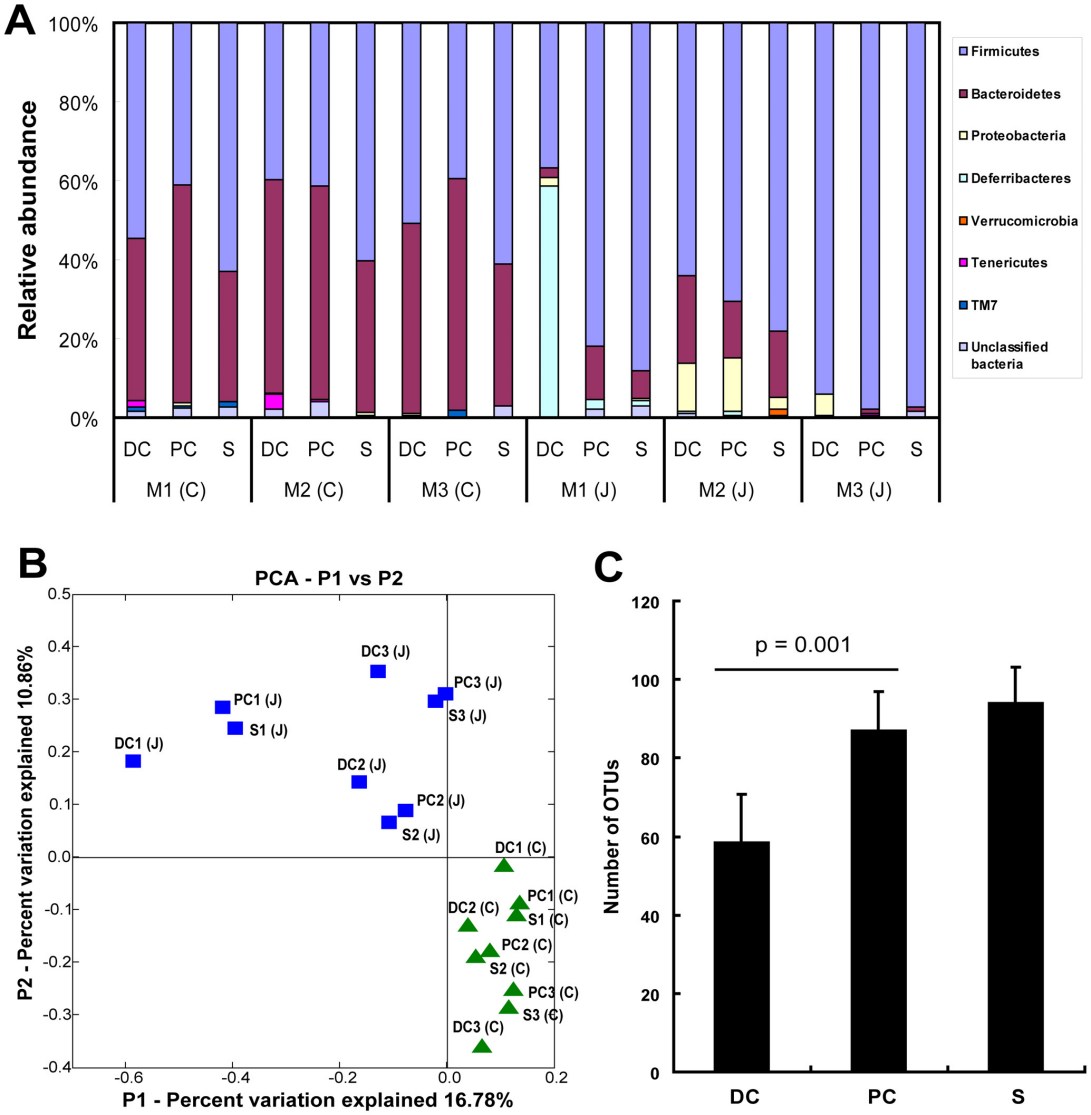
16S rRNA gene clone library sequence analysis of microbial communities in mouse colon and stool samples. (A) Relative bacterial composition in colonic and stool samples from 3 mice (M1–M3) bred at the University of Chicago (C) and 3 obtained from the Jackson laboratories (J) were shown at the phylum level. 16S rRNA gene sequences were grouped into different phyla using the RDP classifier tool at a default confidence threshold (80%). (B) Principal coordinate analysis (PCA) of clone libraries was performed on samples collected from mice of the University of Chicago (triangles (green)) and mice obtained from the Jackson Laboratories (squares (blue)). Mice from the two different facilities clustered separately. Clustering of distal colon (DC) samples was also distinct from those of the proximal colon (PC) and feces (S) in each individual mouse. (C) OTUs were assigned and calculated by DOTUR analysis at 97% cutoff level. Fewer OTUs were found in mouse distal colon compared to those found in the proximal colon and feces.

To gain insight into the specific bacterial phylotypes of the mouse proximal colon, distal colon and feces, 16S rRNA gene clone libraries were established and sequenced. The regional colonic microbiota among different litters was also examined to assess whether observed patterns in regional microbiota were random or reproducible. Two groups of conventional WT C57BL/6J mice were studied: (1) mice bred and housed in the animal care facility at the University of Chicago, and (2) mice bred and shipped from the Jackson Laboratory and maintained separately in the animal care facility. Samples from a representative mouse from each of 3 different litters of the Chicago (M1–3 (C)) and Jackson Laboratory (M1–3 (J)) groups were then harvested to construct the 16S rRNA gene libraries (Figure 2). A total of 3059 partial 16S rRNA gene sequences (average sequence length of 650 nucleotides) were obtained. 16S rRNA gene sequences were assigned into operational taxonomic units (OTUs) or phylotypes at a similarity cutoff value of 97%, such that sequences which were more than 97% similar were considered the same. Library coverage was calculated by Good's formula, and ranged from 70% to 80%, indicating that the 16S rRNA gene sequences from each library encompassed the majority of the bacterial population in the sample. Using the RDP Classifier tool to assign taxonomy to each 16S rRNA gene sequence, the majority of sequences were classified into two phyla *Firmicutes* and *Bacteroidetes*. *Proteobacteria*, *Deferribacteres*, *Verrucomicrobia*, *Tenericutes*, *TM7* and a small portion of unclassified bacteria were represented at lower levels in most of the samples except for M1 (J) which had a relative dominance of *Deferribacteres* in the distal colon (Figure 2A). A small percentage of sequences belonging to this phylum were also found in all the samples harvested from mice obtained from the Jackson laboratory (less than 3%, Figure 2A). However, *Deferribacteres* could not be detected in the 1525 sequences collected from animals bred in the Chicago animal facility.

Further analysis of the partial 16S rRNA gene sequences that were identified as *Deferribacteres* showed that a total of 126 sequences in 9 samples could be assigned to 4 OTUs (based on 97% sequence identity). Ten colonies from each representative OTU were picked and further sequenced to obtain near full-length 16S rRNA gene sequences (accession numbers: FJ042492 to FJ042495). BLAST analysis, these sequences shared a close similarity (99%) with the previously described species *Mucispirillum schaedleri* which was first identified in mice from The Jackson laboratory. A phylogenetic tree was constructed from a multiple sequence alignment of 1510 bp 16S rDNA gene sequences using the tree builder tool in RDP. It included the four representative sequences from the aforementioned 4 OTUs identified in this study and 4 representative sequences available in the NCBI GenBank database described as *Mucispirillum schaedler*, as a control (Figure S1). The high degree of consensus and close relative phylogenetic position among these sets of sequences indicated that these 4 identified phylotypes fall into the same genus as *Mucispirillum schaedleri* with high confidence.

Similar to other reports, two bacterial phyla [13], *Firmicutes* and *Bacteroidetes*, were dominant in the mouse enteric microbiota. However, there was a clear difference in the relative representation of these phyla in mice bred at the two different facilities. Mice bred at The University of Chicago had a significantly lower proportion of *Firmicutes* and higher proportion of *Bacteroidetes* (F: 50.1%; B: 46.5%) compared to mice from Jackson laboratory (F: 78.4%; B: 8.9%; p<0.01). This can be further appreciated by the principal coordinate analysis (PCA) shown in Figure 2B and UniFrac P-Test (Table S1) where the separation of sample microbiota, regardless of regional location, could be seen between the two groups of mice.

However, despite the differences in microbiota among the various litters, regional differences between the mucosa-associated microbiota of distal colon versus proximal colon and feces remained consistent. This is shown in Figure 2C and Figure S2 where the T-RFLP results of two different litters are compared. While the microbiota differed substantially between the litters, the distal colon microbiota consistently clustered separately from the proximal colon and feces which were similar to each other. Further analysis of the differences of microbiota between mouse proximal and distal colon did not reveal a specific pattern of microbial communities shared among litters. However, limited diversity was invariably found in distal colon of all mice by 16S rRNA gene library cloning and sequencing as demonstrated by T-RFLP. The library cloning showed that the distal colon had significantly lower numbers of OTUs compared to the proximal colon and stool sample (Figure 2C, p = 0.001). This conclusion was also supported by rarefaction analysis in each individual mouse (Figure S3).

### Differential expression of TLR2 and TLR4 in mouse colonic mucosa

**Figure 3:**
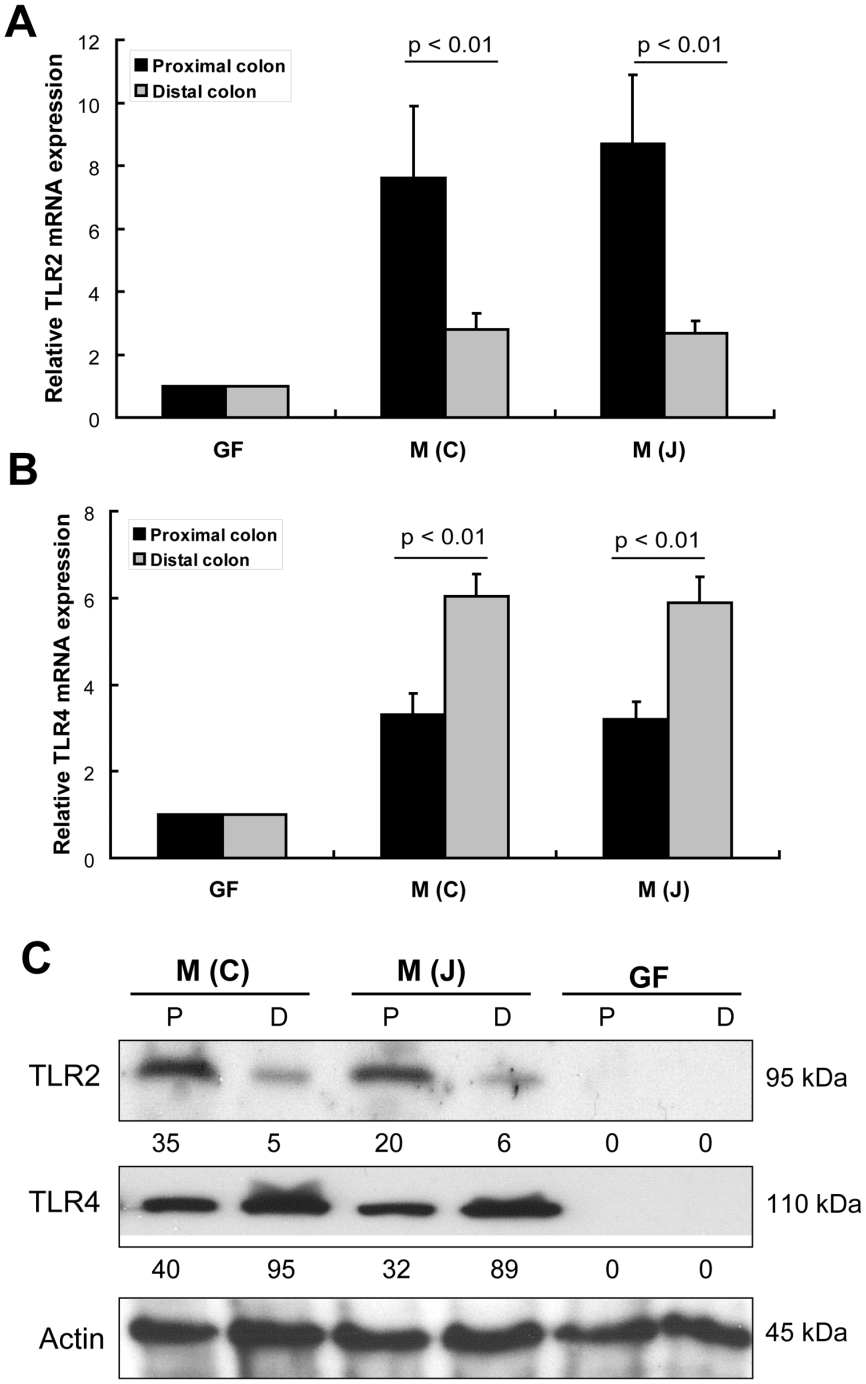
Expression of TLR2 and TLR4 in mouse colon. (A) and (B) Colonic mucosal scrapings from SPF and GF mice were used to extract RNA. Expressions of TLR2 and TLR4 between mouse proximal colon and distal colon were compared by real-time PCR as described in Methods (n = 12). (C) Expression of TLR2 and TLR4 in mucosal scrapings by Western blot. Cell lysates from mucosal scrapings of mouse proximal colon (P) and distal colon (D) were separated on a 10% SDS-PAGE gel and blot by anti-TLR2 and anti-TLR4 specific antibodies respectively. Relative protein expression was quantified by measuring the densitometry using NIH Image J 1.54 software which was denoted below each sample. Expression of actin was used as an internal control for each sample.

**Figure 4:**
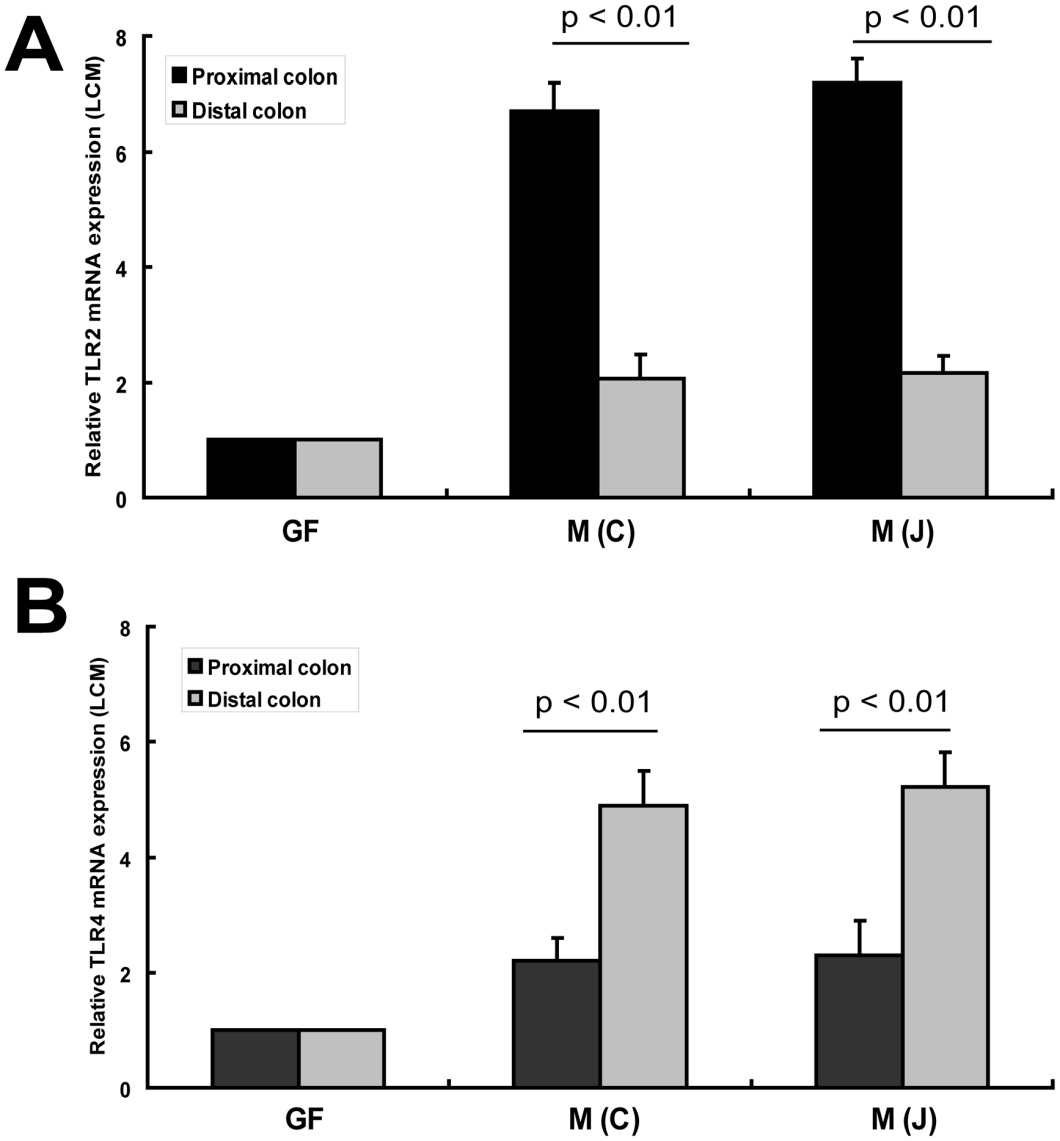
Analyses of expression of TLR2 and TLR4 in mucosal epithelial cells by LCM. Laser capture microdissection (LCM) was performed on frozen sections of mouse proximal colon and distal colon. RNA was extracted from microdissected mucosal epithelial cells and transcribed into cDNA which were used to PCR-amplify TLR2 and TLR4, respectively. The expression of GAPDH was used as an internal control. (A) and (B) Quantitative real-time PCR was used to compare the mRNA expression of TLR2 and TLR4 in microdissected mucosal epithelial cells as described in Methods (n = 6).

Toll-like receptors (TLRs) play a key role in innate immunity by regulating regional host immune responses, and, in doing so, we believe affects assemblage of mucosa-associated microbiota. We therefore examined the mucosal expression of TLR1, TLR2, TLR3, TLR4 and TLR5 in proximal and distal colon using real-time PCR. These TLRs were selected because they have been reported to be expressed in colonic epithelial cells [14], [15]. As shown in Figure S4, transcriptional expression of those TLRs was compared between mouse proximal and distal colon in both SPF mice and GF mice. Increased expression of TLRs was found in conventional WT mice compared to their GF controls. However, only TLR2 and TLR4 were found to be differentially expressed in the colon of SPF C57BL/6J mice (Figure 3). Both RT-PCR and Western blot analysis showed that TLR2 was highly expressed in proximal colon while TLR4 was highly expressed in mouse distal colon (Figure 3). In contrast, TLR2 and TLR4 were minimally expressed in the colonic mucosa of GF mice by Western blot analysis (Figure 3C). The regional differences in TLRs in the mouse colon are therefore likely dependent upon the regional differences of mucosa-associated bacteria. This analysis was from mucosal scrapings that may contain many cell types that express TLRs, therefore, to determine if the differential expression of TLR2 and TLR4 was specific to the colonic epithelium, laser capture and microdissection were performed to harvest colonic epithelial cells from proximal and distal colon in both SPF and GF mice. After RNA extraction from colonic epithelial cells, transcriptional expression of TLR2 and TLR4 in colonic epithelial cells of GF mice were detected by reverse transcriptional PCR and gel electrophoresis (data not shown here). However, the differential transcriptional expression of TLR2 and TLR4 along the proximal-distal axis of the colon was only found in microdissected epithelial cells of SPF mice but not in those of GF mice by real-time PCR (Figure 4). Thus, the responses in TLR expression to the enteric microbiota of whole mucosa are reflected by changes in colonic epithelial cells in these regions.

### GF mice and microbiota transplantation

**Figure 5:**
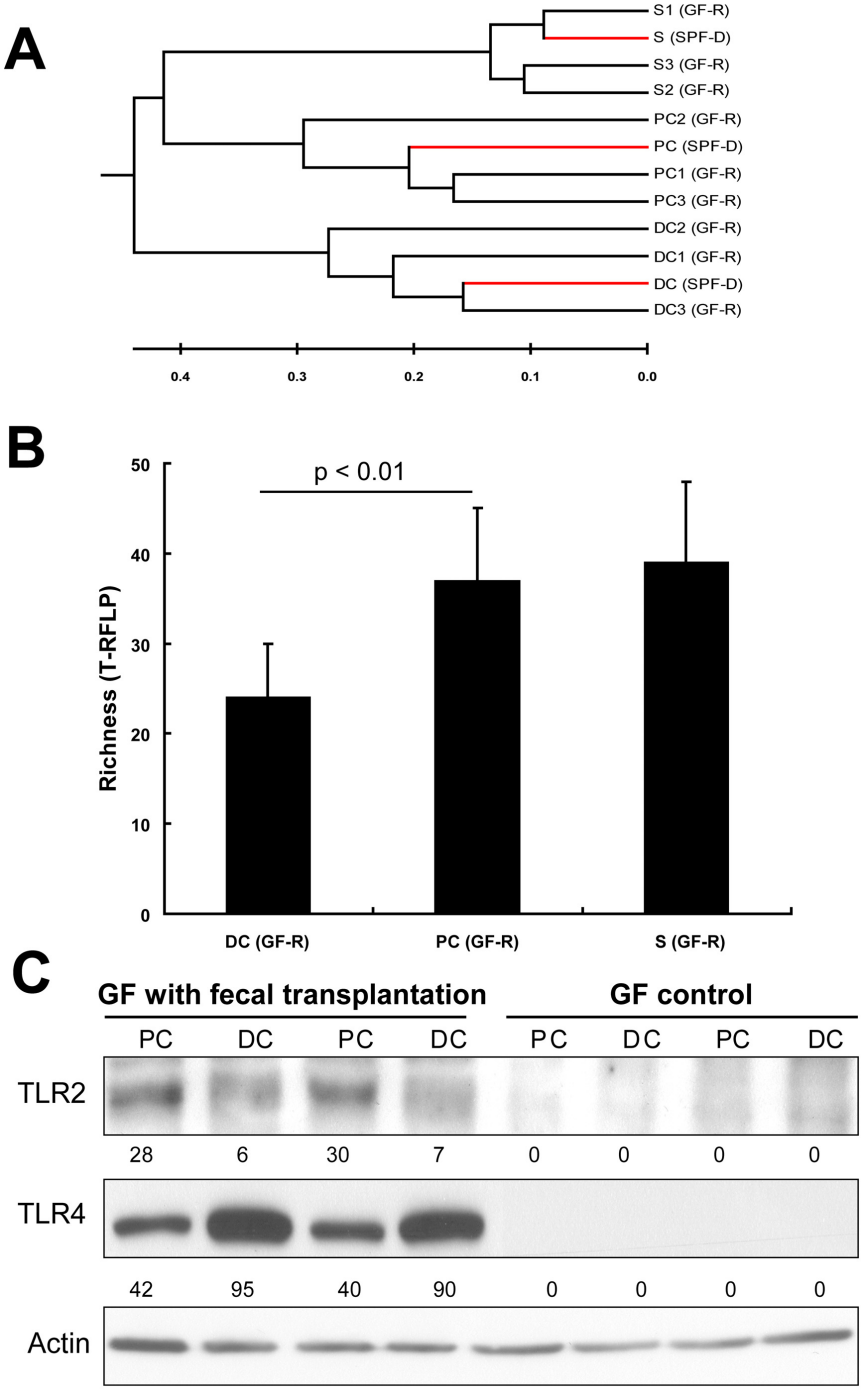
Microbiota transplantation (transfaunation) in germ-free (GF) mice. GF mice on C57Bl/6J background were transferred with cecal content of SPF mice by gavage. After 3 weeks colonization, mice were sacrificed and colonic tissues and stool samples were collected for bacterial and gene expression analysis. (A) Microbiota in stool (S), proximal colon (PC) and distal colon (DC) of GF recipient mice (GF-R) was established after 3 weeks transplantation. The T-RFLP profiles are similar between donor (SPF-D, n = 1) and recipient mice (n = 3) for corresponding stool and tissue samples. Proximal colon and distal colon clustered differently in transfaunated GF recipient mice. Similar to donor mice, the richness of the distal colonic microbiota was less than that of proximal colon and stool (B). The regional expression of TLR2 and TLR4 (C) was restored in GF mice following transfaunation. Relative protein expression was denoted below each sample. Actin was used as the internal loading control.

To determine the role of enteric microbiota in regulating regional TLR2 and TLR4 expression, microbiota transplantation (transfaunation) experiments were performed using GF C57Bl/6J mice. Microbiota from the cecum of conventional SPF WT mice were prepared as previously described [16] and transferred into GF recipient mice by oral gavage (200 ul cecal content in sterile saline buffer). Three weeks post-colonization, GF control mice and GF mice transfaunated were terminated. Samples from stool and proximal and distal colons were harvested for bacterial analysis. By T-RFLP analysis, both stool and mucosa-associated microbiota were established in GF mice with microbiota transplantation. T-RFLP profiles from stool, proximal and distal colon in GF recipient mice clustered tightly with the profiles of corresponding site samples from donor mice (Figure 5A). As in the SPF donors, the regional mucosa-associated microbiota of proximal and distal colon was significantly different. The mucosa-associated microbiota or the distal colon had a much lower diversity compared with that in proximal colon (Figure 5B). These findings suggest that regional host factors determine the assemblage of regional mucosa-associated microbiota.

Following transfaunation, the regional pattern of TLR2 and TLR4 expression in colonic mucosa was restored, as assessed by real-time PCR (data not shown) and Western blot analysis (Figure 5C). These results support the notion that the mucosal expression TLR2 and TLR4 in murine colon is dependent on regional mucosa-associated microbiota which is important in maintaining the gut innate immune function of the host.

### Induction of TLR2 expression in distal colon upon exposure to cecal microbiota

**Figure 6:**
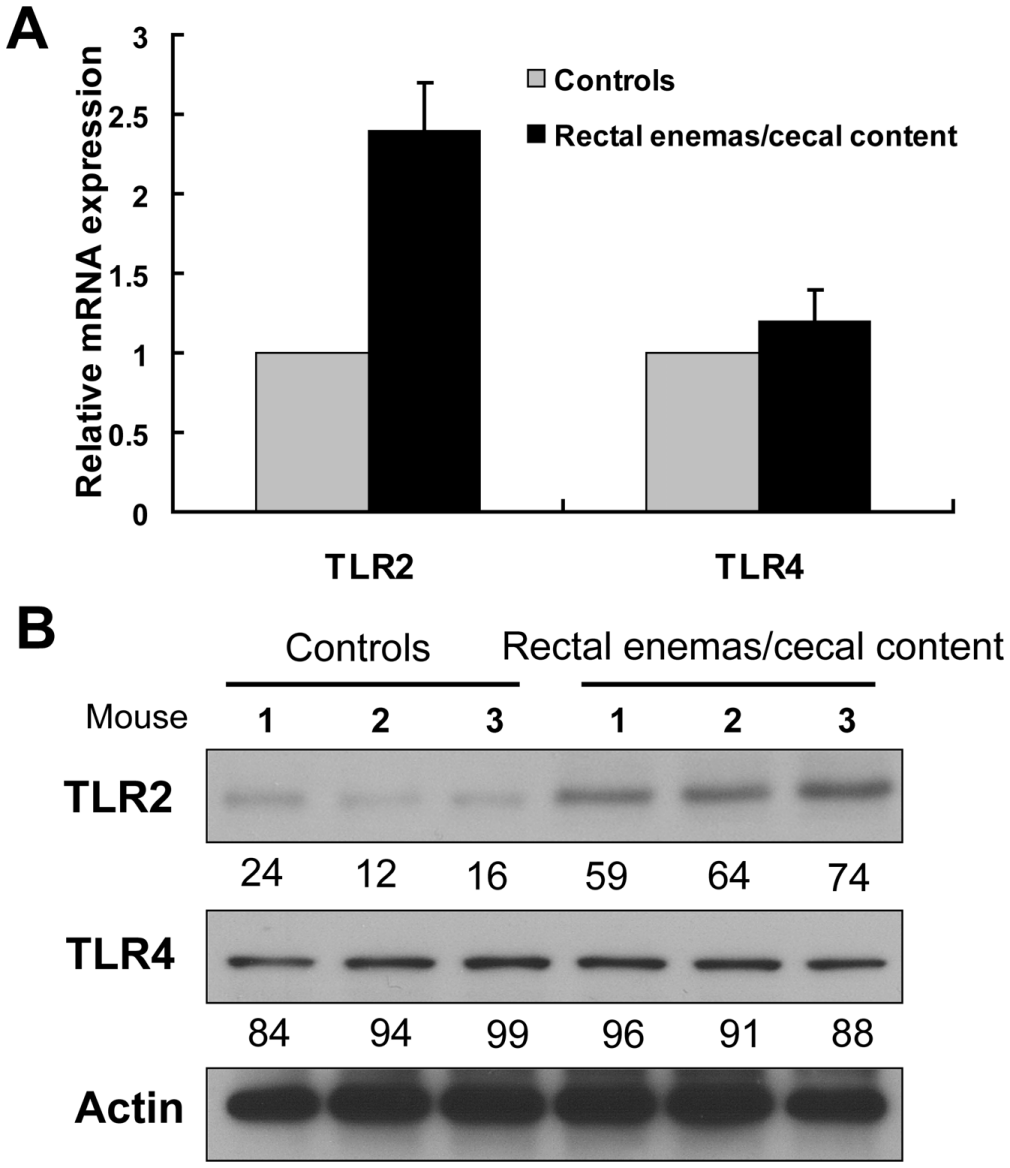
Transrectal administration of cecal microbiota into the distal colon stimulates TLR2 expression. Mice on C57Bl/6J background housed in SPF conditions were treated by rectal enemas with sterile saline or cecal content prepared as a slurry. Expression of TLR2 and TLR4 in distal colon was compared by real-time PCR (A) and Western blot (B) analysis. Relative protein expression assessed by densitometry was denoted below each sample. Actin was used as the internal loading control (n = 3).

To test whether the differential expression of TLR2 and TLR4 in the colonic mucosa is determined by the regional distribution of mucosa-associated microbiota, rectal enemas were performed using cecal microbiota which had been shown to be very similar to the mucosa-associated microbiota in the proximal colon. After twice daily treatments for 3 consecutive days, we observed the induction of TLR2 expression in the distal colon by exposure of the mucosal surface with the cecal slurry, but not with sterile saline (Figure 6). A major difference between the cecal slurry and luminal pellets of stool in the distal colon is that the former is dispersed and more likely to directly interact with the mucosa. TLR2 expression was increased by more than 3-fold compared to controls by Western blot analysis. In contrast, we did not detect changes in distal colonic TLR4 expression in either group (Figure 6). These data suggest that the mucosa-associated microbiota of the distal colon was still present, but the augmentation of microbial signals from exposure to proximal microbiota was sufficient to induce TLR2 expression as well. Thus, the distal colonic mucosa is not restricted in its ability to mount a TLR2 response and that regional TLR2 and TLR4 expression is most likely determined by the regional heterogeneity in mucosa-associated microbiota.

## Discussion

Host-microbial interactions in the mammalian colon are complex and incompletely understood, yet they play a central role in maintaining intestinal and immune homeostasis and providing the nimbleness to respond to physiological anomalies [1], [2]. For host-microbial interaction to occur, a dynamic mutualistic relationship must exist between host and microbes, where assembly and function of specific microbes are modulated by host factors and vice versa to achieve a state of stability and mutual benefit. In this study, we demonstrate an almost inseparable relationship between mucosa-associated microbiota and mucosal TLR expression. We observed, for instance, that cecal microbiota transferred to germ-free recipient mice can fully restore the pattern of regional mucosa-associated microbiota, a finding that has several important implications. First, cecal microbiota, which has the greatest diversity of the GI tract, is the source for most, if not all mucosa-associated microbiota of the proximal and distal colon. Second, as the cecal microbiota move distally, local conditional factors play a major role in determining the assembly rules that dictate which microbes are selected to become part of the more stable community of mucosa-associated microbiota. In the proximal colon, the host factors are similar to those in the cecum, as the microbiota in these two regions cluster closely. In contrast, host conditions of the distal colon are different, resulting in a less diverse mucosa-associated microbiota that cluster separately from those found in the cecum, proximal colon, distal colonic lumen and stool. Examples of host factors that can determine region-specific differences in mucosa-associated microbiota mucus composition, nutrient availability, motility, blood flow, and immune factors such as the regional expression of TLRs in both immune and epithelial cell compartments.

We found the profile of mucosa-associated microbiota from the proximal colon was relatively similar to feces in the rectal vault (essentially luminal microbiota of the distal colon). Thus, as stool forms in the transverse colon, most of these microorganisms are retained and sequestered in the stool. There appears to be little contribution to stool microbes by distal colonic mucosa-associated microbiota and vice versa. While the enteric microbiota of these regions varied considerably among different litters of in-house and Jackson Laboratory bred mice, the clustering of regional patterns remained consistent, i.e. proximal colonic mucosa-associated and rectal vault stool samples were similar, but both were significantly different from that in the distal colon. Analysis of microbiota richness showed that distal colonic mucosa-associated microbiota was consistently less diverse than that of proximal colon, cecal.

Once assembled, the mucosa-associated microbiota of the proximal and distal colon play an important role in regulating host gene expression and cellular functions [17]. Our data support this notion because the exposure of the distal colonic mucosa to cecal microbiota induces TLR2 expression. Thus, the gradients of TLR2 and TLR4 expression along the length of the colon are not caused by inherent limitations in tissue response to microbial stimulus. Rather, it appears that regional TLR2 and TLR4 expression are determined by the overlying mucosa-associated microbiota.

Our finding of differences in regional TLR2 and TLR4 expression along the length of the colon confirm observations by Kinoshita and colleagues [18]. However, similar patterns of expression are found among several other host genes whose expression are regulated by microbial signals, including peroxisome proliferator-activated receptors [19], TGF-B activators [20], inducible heat shock proteins (unpublished data) and vitamin D receptor [21]. Many of these genes are involved in the regulation of immune/inflammatory responses, cell growth and differentiation, cytoprotection, and barrier function in the mammalian colon. Their region-specific expression is likely due to differences in region-specific mucosa-associated microbiota, but also imparts specialized functions that are inherently important to host physiology or maintenance of intestinal homeostasis.

While this study does not address the purpose of region-specific TLR expression, we offer two possibilities for consideration. First, the microbiota-induced expression profile of TLRs may be a self-reinforcing mechanism to further stabilize the existing mucosa-associated microbiota or deter non-compatible microbes from colonization through negative selection. In support of this possibility is the observation that NOD mice deficient in MyD88 gene expression have a distinct colonic microbiota profile from their heterozygote counterparts [22], ultimately influencing the clinical outcome of experimental type I diabetes. Another possibility for the region-specific, microbe-dependent TLR expression is that TLR2 can dimerize with either TLR1 or TLR6, resulting in skewing of naïve T cells towards either an immunogenic or tolerating immune response [23]. Thus, the greater expression of TLR2 in the proximal colon may provide a more adaptable immune response capable of dealing with a microbial population of greater diversity and abundance.

Regional differences in colonic microbiota and microbiota-dependent host gene expression could also influence the pattern and presentation of certain bowel diseases. Inflammatory bowel diseases, for instance, have very characteristic presentations and patterns to their mucosal inflammation, suggesting a role for topical factors such as mucosa-associated microbiota. Among different individuals, for example, the extent of ulcerative colitis can vary, but the disease invariably starts in the rectum. A similar pattern of inflammation is seen in DSS-induced experimental murine colitis where the inflammation is predominantly found in the distal colon and rectum [18], [24], [25]. We speculate that the composition and less diverse nature of the distal colonic mucosa-associated microbiota could favor the emergence of disease-promoting microbes. Alternatively, we raise the possibility that the declining proximal-distal gradient of host factors important for maintaining immune and intestinal homeostasis (e.g. TLR2, heat shock proteins, vitamin D receptor) could render the distal colon more susceptible to injury and the development of ulcerative colitis.

In summary, we find regional heterogeneity in mucosa-associated microbiota and TLR2 and TLR4 expression which appear to be interdependent. The findings also underscore a specific role of mucosa-associated microbiota that is distinct from the more transient population of luminal microbiota. Furthermore, our study raises the question as to whether the prevailing practice of sampling just luminal or stool microbiota is sufficient or appropriate for understanding the role of the enteric microbiome in health and disease. Finally, we believe the region-specific differences in mucosa-associated microbiota and their dependent host gene expression play an important role in contributing to the specific physiological functions of different parts of the mammalian colon.

## Materials and Methods

### Animals and samples collection

Male, conventional wild type (WT) C57BL/6J mice (8–10 w/o) were maintained in a 12-h light:dark cycle and allowed free access to food and water. Male, germ-free WT C57BL/6J mice (8–10 w/o) were maintained under the same conditions in the gnotobiotic facility of the Digestive Disease Research Core Center (DDRCC) at the University of Chicago. The germ-free (GF) colonies are routinely tested for microbes and parasites by the facility's staff and by our lab to ensure germ-free conditions. Two groups of conventional WT mice were studied. One was bred and housed in the animal care facilities of the University of Chicago under specific pathogen free conditions (SPF), while the other was obtained from the Jackson Laboratories and housed separately in the animal care facility. All groups of mice were allowed ad-libitum access to Harlan Teklad 7012 (SPF) or its autoclaved equivalent NIH 31 (GF) chow. All animal studies were approved by the Institutional Animal Care and Use Committee of the University of Chicago (permit number 72007).

Colonic samples at 1 cm proximal to the colonic-anal junction and distal to the cecal-colonic junction, respectively, were harvested for analysis. These sites will be referred to as distal and proximal colon, respectively. For bacterial population analysis, approximately 0.5 cm intact proximal colon and distal colon devoid of macroscopic luminal content were collected with minimal manipulation. Additional samples of the luminal contents of the cecum and colon were obtained as stool samples. All samples were kept on ice until completion of the tissue harvest and then immediately processed.

### DNA extraction, PCR and terminal restriction fragment length polymorphism (T-RFLP)

Samples were first homogenized in 1 ml extraction buffer [50 mM Tris (pH 7.4), 100 mM EDTA (pH 8.0), 400 mM NaCl, 0.5% SDS] containing 20 ul proteinase K (20 mg/ml). 500 ul of a slurry of 0.1-mm-diameter zirconia/silica beads (BioSpec Products, Bartlesville, OK) were added into the extraction tubes and a Mini-Beadbeater-8k Cell Disrupter (BioSpec Products) set on 5 min was used to lyse the microbial cells. After overnight incubation at 55°C, standard DNA extraction with phenol:chloroform:isoamyl alcohol, and precipitation with ethanol was performed. Isolated DNA was dissolved in TE buffer and stored at −80°C.

Bacterial populations in the samples were analyzed by 16S rRNA gene sequence-based T-RFLP as described previously [26]. Briefly, 16S rRNA gene sequences were amplified from DNA templates using broad-range primers 8F (5′-AGAGTT TGATCCTGGCTCAG-3′) labeled with 6′ carboxyfluorescein (6-FAM) and 1492R (5′-GGTTACCTTGTTACGACTT-3′) for the bacterial domain. PCR products were verified by electrophoresis of aliquots of PCR mixtures (8 ul) in 1.0% agarose and purified by precipitation. Aliquots of purified PCR products were digested by 20 U *Msp* I (New England Biolabs Inc.) and subsequently subjected to capillary electrophoresis using the Applied Biosystems DNA sequencer 3130.

Restriction-digest fragment abundance was determined using GeneMapper software (Applied Biosystems). Raw electropherograms were analyzed for artifacts, such as electrical anomalies, optical cross-talk between the capillaries, baseline drift, fluorescence of non-FAM-labeled contaminants, and distortions of the sizing ladder. Terminal restriction fragment data generated by GeneMapper were filtered and binned by the method developed by Abdo et al [27]. Based on the normalized T-RFLP profile, the number and height of peaks were treated as number and abundance of bacterial phylotypes represented in samples as described previously [26]. Pairwise Bray-Curtis distances [28] were calculated to examine the relationship between communities using the software package MEGA (http://www.megasoftware.net).

### 16S rRNA gene sequence library cloning and sequencing

Unlabeled PCR primers 8F and 1492R were used to amplify 16S rRNA gene sequences from the samples using the same protocol as those for T-RFLP analysis. PCR products were purified by QIAquick gel extraction kit (Qiagen, Valencia, CA) and cloned into pCR-2.1-TOPO® vectors (Invitrogen, Carlsbad, CA) using the TOPO-TA Cloning Kit according to the manufacturer's instructions. From each library, 200 colonies were picked randomly and processed for DNA sequencing using 8F as the primer.

### Sequence alignment and phylogenetic analysis

The 16S rRNA gene sequences were analyzed as described previously [26]. Briefly, raw sequence data were processed using the RDP pipeline server at the Ribosomal Database Project II (RDP-II) website (http://rdp.cme.msu.edu/pipeline) by base-calling, quality-trimming and alignment. Potential chimeric sequences were checked using the SimRank 2.7 package available through the RDP and excluded [29]. The classifier analysis tool of RDP-II and NEBI BLAST tool were used to assign 16S rRNA sequences to the taxonomical hierarchy at different levels. The program DOTUR with the furthest neighbor algorithm was used to group sequences into operational taxonomical units (OTUs) or phylotypes which represented the number of 16S rRNA sequence similarity groupings. A 97% cutoff value was used such that sequences with more than 97% similarity were considered the same. For principal coordinate analysis (PCA), all 16S rRNA gene sequences were imported into the ARB software package and aligned into a phylogenetic tree which was used to perform clustering analysis using online UniFrac without abundance weighting [30]. The P-Test in the UniFrac was performed to determine whether each sample was significantly different from others. All sequences were deposited in the GenBank nucleotide sequence databases under the accession numbers HQ318942-HQ322000.

### RNA extraction, reverse transcription and quantitative real-time PCR

Total RNA was extracted from mouse colonic mucosal scraping by Trizol (Invitrogen, Grand Island, NY) according to the manufacturer's instructions. Complementary DNA was synthesized using SuperScript II (Invitrogen) and random hexanucleotide primers. The forward and reverse primers to amplify TLRs were listed in Table 1. Real-time PCR was performed in an iCycler (Bio-Rad, Hercules, CA) using iQSYBR Green PCR Supermix (Bio-Rad). A two-step quantification cycling protocol was used. The Ct value is defined as the cycle number at which the fluorescence crosses a fixed threshold above the baseline. As a relative quantification, fold changes were measured using the ΔΔCt method as described previously [31].

**Table 1:** Primers used for real-time PCR.

Gene name	Forward primer	Reverse primer	Product size (bp)
TLR1	ATGGGGGAATCCCATGCGCC	TGCAAAGCCTGCAGGTGGGTG	172
TLR2	GCTGGAGGACTCCTAGGCT	GTCAGAAGGAAACAGTCCGC	151
TLR3	GCAACCCTTTCAAAAACCAG	CGCAACGCAAGGATTTTATT	142
TLR4	ACCAGGAAGCTTGAATCCCT	TCCAGCCACTGAAGTTCTGA	186
TLR5	ACCACACTTCAGCAGGATCA	AGTTGAAGCTGAGCAGGAGC	191

### Laser capture microdissection (LCM) and Real time (RT)-PCR

The portions of proximal and distal colons were excised, embedded in OCT compound (Sakura Finetechnical, Tokyo, Japan), frozen on dry ice, and the tissue blocks were stored at −80°C. Frozen sections were cut to 6 µm thickness, mounted on positively charged MembraneSlides (PEN-Membrane 2.0 um, Leica), stained with the H&E method for LCM, and then air dried. LCM was performed using a Leica AS LMD system to harvest the mucosal epithelial cells. The microdissected samples were carefully collected into a PCR tube. RNA was extracted by using a PicoPure RNA Isolation Kit (Arcturus, Mountain View, CA) according to the manufacturer's instructions. The RNA was converted into cDNA using SuperScript II and random hexanucleotide primers (Invitrogen). Both regular PCR and real-time PCR were performed to detect the expression of TLRs in the cDNA samples.

### Western blot analysis

Mouse mucosal scraping samples from proximal and distal colons were homogenized in cell lysis buffer (Cell Signaling Technology, Danvers, MA). Protein concentration determination and immunoblotting were performed as previously described [31]. Briefly, twenty micrograms of protein were separated by SDS-PAGE, transferred to polyvinylidene difluoride membranes and blotted with primary antibodies for TLR2, TLR4, and actin (Cell Signaling Technology, Danvers, MA). The specificity of the anti-TLR2 and –TLR4 antibodies was confirmed by Western blot comparison of proteins harvested from colonic mucosa from TLR2- and TLR4-knockout (KO) mice (Figure S5). Quantification of images was performed by scanning densitometry using NIH Image J 1.54 software (National Institutes of Health, Bethesda, MD).

### Microbiota transplantation (transfaunation) experiments

Germ-free WT C57Bl/6J mice (8–10 w/o) were gavaged orally with cecal luminal contents harvested from C57Bl/6J donor mice (transfaunation) housed in the SPF facility (1 donor and 3 recipients; 2 independent experiments). Donor mice were terminated at day 0 and recipient mice 21 days after transfaunation. Cecal contents and colonic samples from both donor and recipient mice were collected for microbiota analysis as described previously [16].

### Rectal enemas with cecal contents

Littermate conventional C57Bl/6J mice were randomly divided into a control or treatment group (n = 3 each). One additional mouse from the same litter was terminated at day 0 as the donor and the cecal content were collected, re-suspended in 4 ml sterile saline and stored in −20°C. Each morning and afternoon for 3 consecutive days, mice were anesthetized by an intraperitoneal injection of 200 µl of a mixture of 10 mg/ml of ketamin (LIoyd, Shenandoah, IA) and 1 mg/ml of xylazine (Abbott laboratories, Chicago, IL). Mice then received a rectal instillation of 200 µl of saline or 200 ul of a cecal slurry in saline by 2.4 cm steel cannula for control mice or treatment mice, respectively. Mice were kept in a head down vertical position for 20 min before returning to their cages to optimize retention of the enema solution. Following the last treatment on day 3, all mice were sacrificed and mucosal scrapings from the distal colon collected for analysis.

### Statistical analysis

All data were expressed as mean ± SD. Student's t-test and one-way ANOVA were used to test the significance of differences between groups or samples. Statistical significance was set at p<0.05.

## Supporting Information

Figure S1 Phylogenetic relationships between the operational taxonomic units (OTUs) of Deferribacteres identified in this study and 16S rRNA gene sequences of Mucispirillum schaedleri derived from NCBI GeneBank database. Sequences identified in this study were aligned with 16S rRNA gene sequences of Mucispirillum schaedleri deposited in the GeneBank using the aligner in RDP. A rooted tree was built up by using the Neighbor weighted neighbor-joining tree building algorithm. Scale bar represents 1% sequence divergence. Bootstrap values are based on 100 replications.
(0.24 MB TIF)

Figure S2 Differences of gut microbiota between different mouse litters. Stool sample (S) and proximal colon (PC) and distal colon (DC) were collected from mice from two different litters. Microbial populations in these samples were analyzed by T-RFLP. By Bray-Curtis analysis, corresponding samples of the mucosa-associated microbiota from two separate litters differed significantly (blue versus green). However, within the same litter, proximal colonic mucosa-associated and stool microbiota were very similar, but distinctly different from the mucosa-associated microbiota of the distal colon. The scale bar shows the distance of similarity.
(1.13 MB TIF)

Figure S3 Rarefaction curves for sequences from six mice. 16S rRNA gene sequences of stool sample (S), proximal colon (PC) and distal colon (DC) in each individual mouse were grouped at 97% cutoff level. Sequences sharing the highest similarity to the same phylotype were grouped together under the same OTUs. Number of OTUs was assigned and rarefaction curves were constructed by DOTUR analysis.
(1.50 MB TIF)

Figure S4 Analysis of TLRs expression in colonic mucosa by real-time PCR. Colonic mucosal scrapings from SPF and GF mice were used to extract RNA. Expression of TLR1, TLR2, TLR3, TLR4 and TLR5 between mouse proximal and distal colon was analyzed by quantitative PCR as described in Methods. Transcriptional expression of TLRs in colonic mucosa of GF mice was used as controls (n = 12).
(0.91 MB TIF)

Figure S5 Validation of anti-TLR2 and -TLR4 antibodies using TLR2 and TLR4 knockout mice. Colonic mucosal scraping from wild type, TLR2 knockout mice (which were provided by Dr. Bana Jabri) and TLR4 knockout mice (which were provided by Dr. Cathryn Nagler) were used to extract proteins. Expression of TLR2 and TLR4 were analyzed by Western blot. Actin was used as internal control (n = 2 for each group).
(2.20 MB TIF)

Table S1 Comparison results of paired samples by P-Test in UniFrac. The 16S rRNA gene sequence composition between libraries was compared by using the P-Test in the UniFrac. The P values shown in the table as 0.00 mean there is a significant difference between two compared libraries. Differences between two libraries which were not significant were labeled in red.
(0.07 MB DOC)
