# Supplementary figures and images for: Regional Mucosa-Associated Microbiota Determine Physiological Expression of TLR2 and TLR4 in Murine Colon
Source: PLoS One. 2010 Oct 22;5(10):e13607. doi: 10.1371/journal.pone.0013607 (PMC2962643; doi:10.1371/journal.pone.0013607)

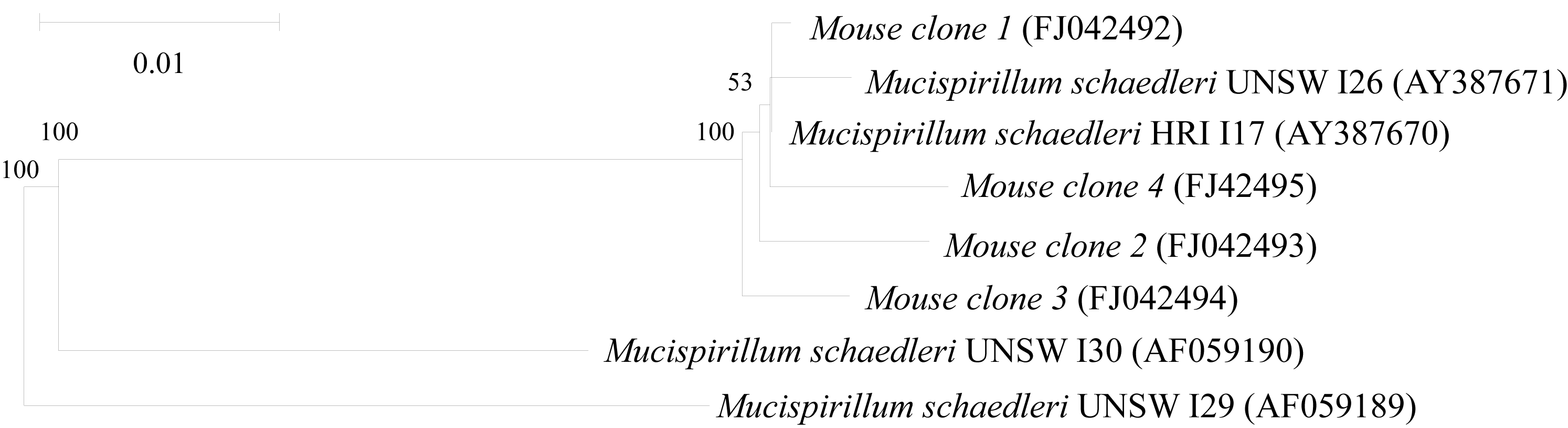

Supplement: Figure S1 — Phylogenetic relationships between the operational taxonomic units (OTUs) of Deferribacteres identified in this study and 16S rRNA gene sequences of Mucispirillum schaedleri derived from NCBI GeneBank database. Sequences identified in this study were aligned with 16S rRNA gene sequences of Mucispirillum schaedleri deposited in the GeneBank using the aligner in RDP. A rooted tree was built up by using the Neighbor weighted neighbor-joining tree building algorithm. Scale bar represents 1% sequence divergence. Bootstrap values are based on 100 replications. (0.24 MB TIF) [file pone.0013607.s001.tif]

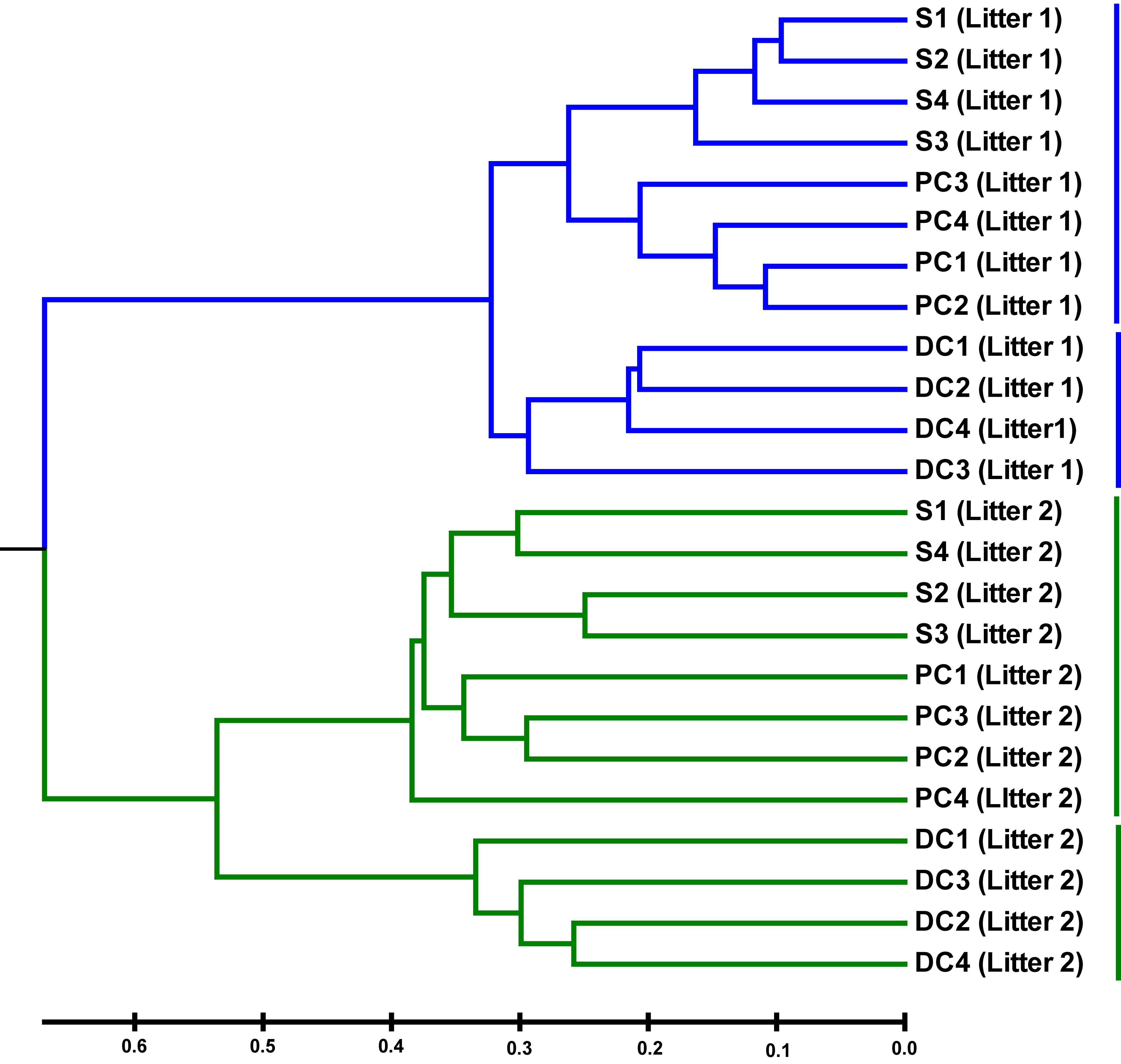

Supplement: Figure S2 — Differences of gut microbiota between different mouse litters. Stool sample (S) and proximal colon (PC) and distal colon (DC) were collected from mice from two different litters. Microbial populations in these samples were analyzed by T-RFLP. By Bray-Curtis analysis, corresponding samples of the mucosa-associated microbiota from two separate litters differed significantly (blue versus green). However, within the same litter, proximal colonic mucosa-associated and stool microbiota were very similar, but distinctly different from the mucosa-associated microbiota of the distal colon. The scale bar shows the distance of similarity. (1.13 MB TIF) [file pone.0013607.s002.tif]

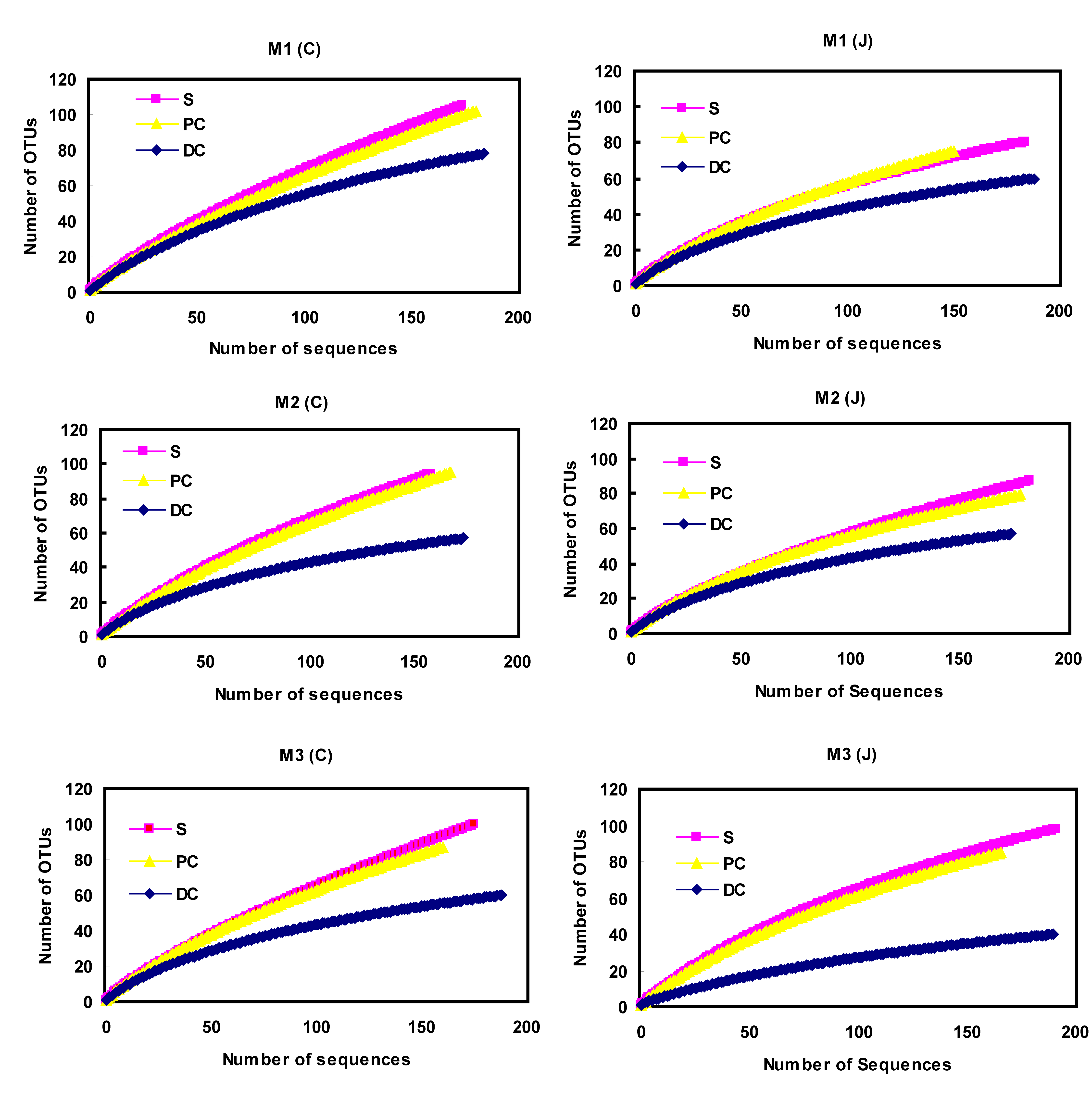

Supplement: Figure S3 — Rarefaction curves for sequences from six mice. 16S rRNA gene sequences of stool sample (S), proximal colon (PC) and distal colon (DC) in each individual mouse were grouped at 97% cutoff level. Sequences sharing the highest similarity to the same phylotype were grouped together under the same OTUs. Number of OTUs was assigned and rarefaction curves were constructed by DOTUR analysis. (1.50 MB TIF) [file pone.0013607.s003.tif]

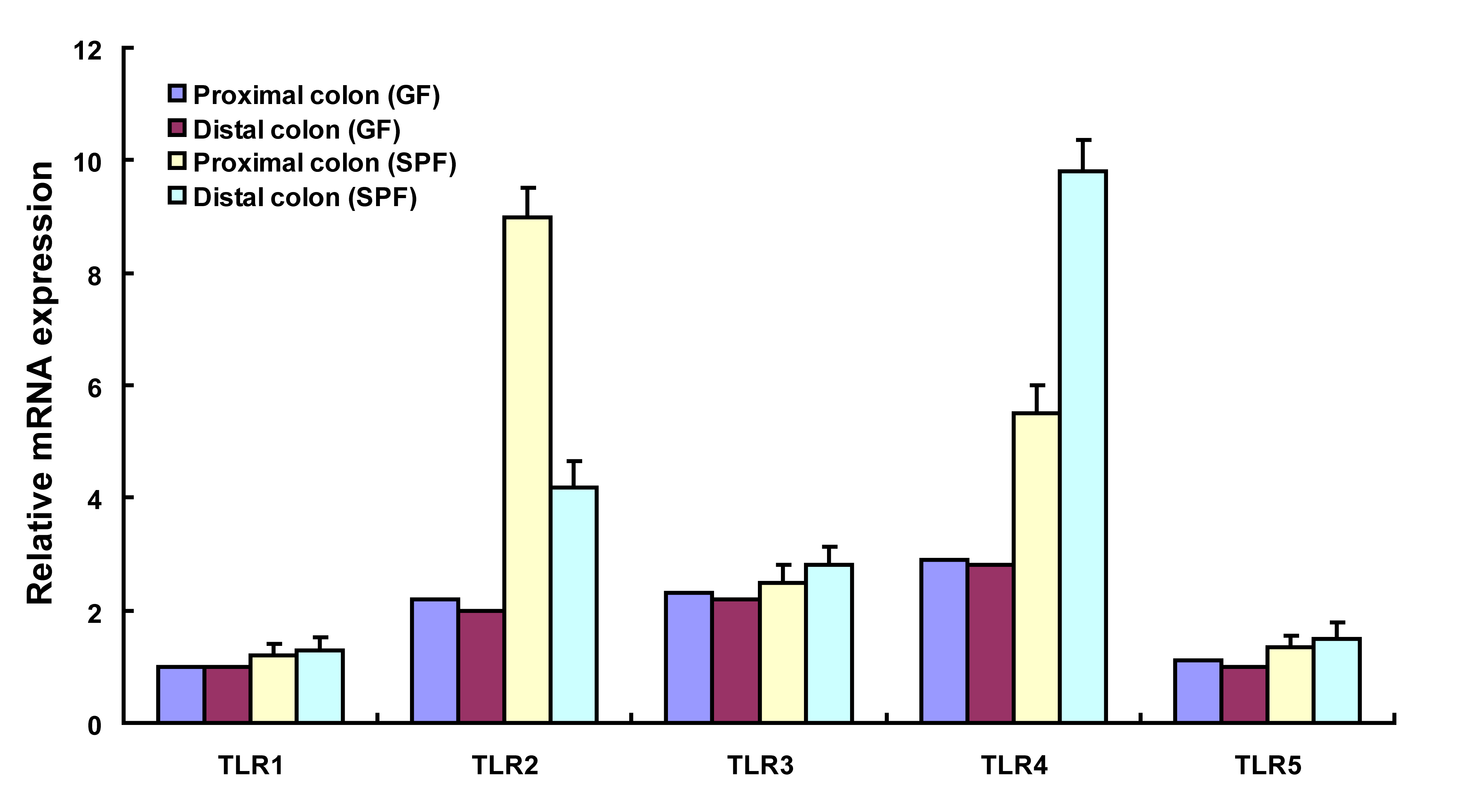

Supplement: Figure S4 — Analysis of TLRs expression in colonic mucosa by real-time PCR. Colonic mucosal scrapings from SPF and GF mice were used to extract RNA. Expression of TLR1, TLR2, TLR3, TLR4 and TLR5 between mouse proximal and distal colon was analyzed by quantitative PCR as described in Methods. Transcriptional expression of TLRs in colonic mucosa of GF mice was used as controls (n = 12). (0.91 MB TIF) [file pone.0013607.s004.tif]

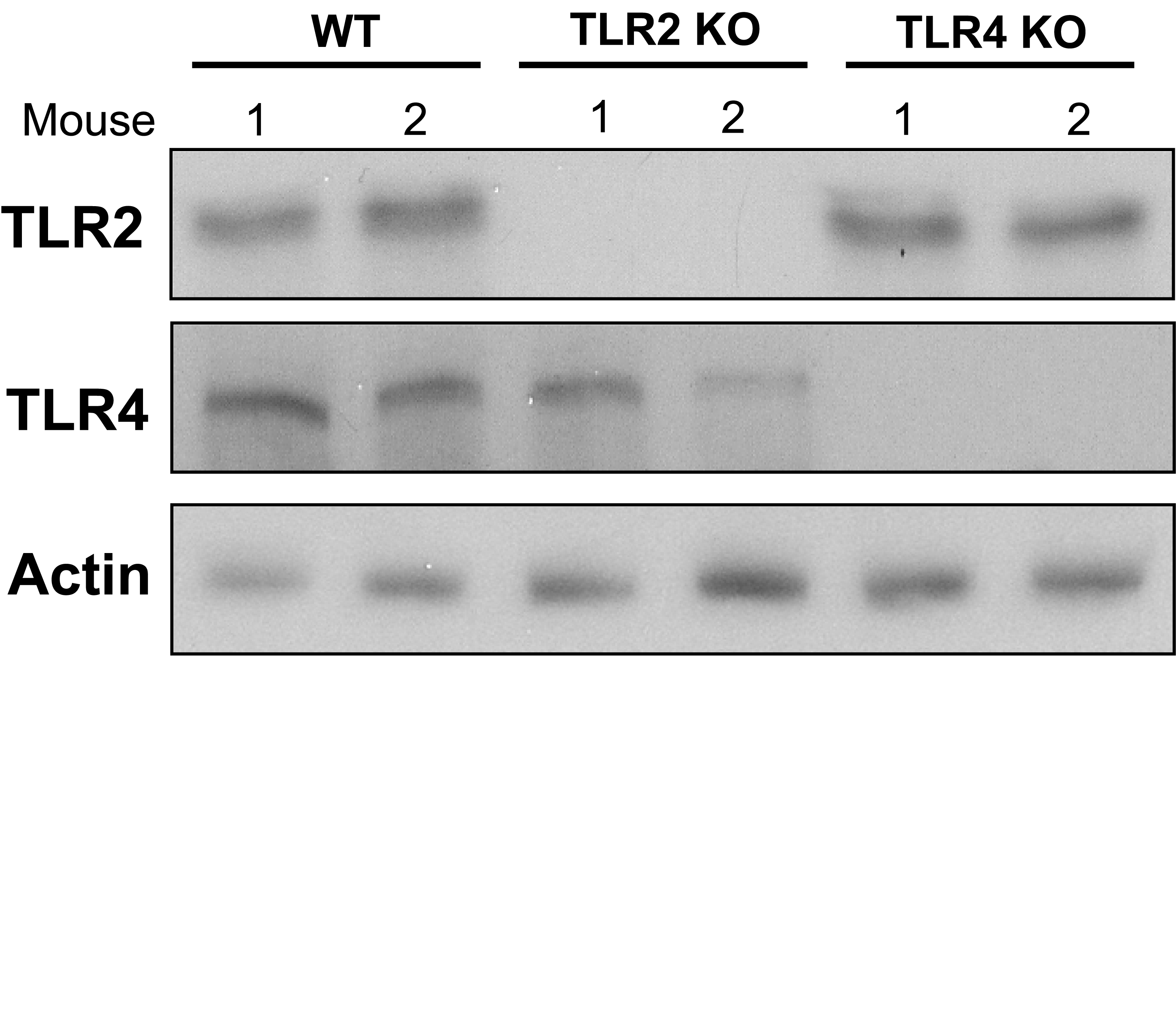

Supplement: Figure S5 — Validation of anti-TLR2 and -TLR4 antibodies using TLR2 and TLR4 knockout mice. Colonic mucosal scraping from wild type, TLR2 knockout mice (which were provided by Dr. Bana Jabri) and TLR4 knockout mice (which were provided by Dr. Cathryn Nagler) were used to extract proteins. Expression of TLR2 and TLR4 were analyzed by Western blot. Actin was used as internal control (n = 2 for each group). (2.20 MB TIF) [file pone.0013607.s005.tif]
