# Supplementary material for: Regional Mucosa-Associated Microbiota Determine Physiological Expression of TLR2 and TLR4 in Murine Colon
Source: PLoS One. 2010 Oct 22;5(10):e13607. doi: 10.1371/journal.pone.0013607 (PMC2962643; doi:10.1371/journal.pone.0013607)
Supplement: Table S1 — Comparison results of paired samples by P-Test in UniFrac. The 16S rRNA gene sequence composition between libraries was compared by using the P-Test in the UniFrac. The P values shown in the table as 0.00 mean there is a significant difference between two compared libraries. Differences between two libraries which were not significant were labeled in red. (0.07 MB DOC) [file pone.0013607.s006.doc]

**Table S1. Comparison results of paired samples by P-Test in UniFrac**

|  | **DC1 (C)** | **PC1 (C)** | **S1 (C)** | **DC2 (C)** | **PC2 (C)** | **S2 (C)** | **DC3 (C)** | **PC3 (C)** | **S3 (C)** | **DC1 (J)** | **PC1 (J)** | **S1 (J)** | **DC2 (J)** | **PC2 (J)** | **S2 (J)** | **DC3 (J)** | **PC3 (J)** | **S3 (J)** |
| --- | --- | --- | --- | --- | --- | --- | --- | --- | --- | --- | --- | --- | --- | --- | --- | --- | --- | --- |
| **DC1 (C)** | - | 0.00 | 0.00 | 0.00 | 0.00 | 0.00 | 0.00 | 0.00 | 0.00 | 0.00 | 0.00 | 0.00 | 0.00 | 0.00 | 0.00 | 0.00 | 0.00 | 0.00 |
| **PC1 (C)** | - | - | 0.21 | 0.00 | 0.00 | 0.00 | 0.00 | 0.00 | 0.00 | 0.00 | 0.00 | 0.00 | 0.00 | 0.00 | 0.00 | 0.00 | 0.00 | 0.00 |
| **S1 (C)** | - | - | - | 0.00 | 0.00 | 0.00 | 0.00 | 0.00 | 0.00 | 0.00 | 0.00 | 0.00 | 0.00 | 0.00 | 0.00 | 0.00 | 0.00 | 0.00 |
| **DC2 (C)** | - | - | - | - | 0.00 | 0.00 | 0.00 | 0.00 | 0.00 | 0.00 | 0.00 | 0.00 | 0.00 | 0.00 | 0.00 | 0.00 | 0.00 | 0.00 |
| **PC2 (C)** | - | - | - | - | - | 0.65 | 0.00 | 0.00 | 0.00 | 0.00 | 0.00 | 0.00 | 0.00 | 0.00 | 0.00 | 0.00 | 0.00 | 0.00 |
| **S2 (C)** | - | - | - | - | - | - | 0.00 | 0.00 | 0.00 | 0.00 | 0.00 | 0.00 | 0.00 | 0.00 | 0.00 | 0.00 | 0.00 | 0.00 |
| **DC3 (C)** | - | - | - | - | - | - | - | 0.00 | 0.00 | 0.00 | 0.00 | 0.00 | 0.00 | 0.00 | 0.00 | 0.00 | 0.00 | 0.00 |
| **PC3 (C)** | - | - | - | - | - | - | - | - | 0.13 | 0.00 | 0.00 | 0.00 | 0.00 | 0.00 | 0.00 | 0.00 | 0.00 | 0.00 |
| **S3 (C)** | - | - | - | - | - | - | - | - | - | 0.00 | 0.00 | 0.00 | 0.00 | 0.00 | 0.00 | 0.00 | 0.00 | 0.00 |
| **DC1 (J)** | - | - | - | - | - | - | - | - | - | - | 0.00 | 0.00 | 0.00 | 0.00 | 0.00 | 0.00 | 0.00 | 0.00 |
| **PC1 (J)** | - | - | - | - | - | - | - | - | - | - | - | 0.08 | 0.00 | 0.00 | 0.00 | 0.00 | 0.00 | 0.00 |
| **S1 (J)** | - | - | - | - | - | - | - | - | - | - | - | - | 0.00 | 0.00 | 0.00 | 0.00 | 0.00 | 0.00 |
| **DC2 (J)** | - | - | - | - | - | - | - | - | - | - | - | - | - | 0.00 | 0.00 | 0.00 | 0.00 | 0.00 |
| **PC2 (J)** | - | - | - | - | - | - | - | - | - | - | - | - | - | - | 0.11 | 0.00 | 0.00 | 0.00 |
| **S2 (J)** | - | - | - | - | - | - | - | - | - | - | - | - | - | - | - | 0.00 | 0.00 | 0.00 |
| **DC3 (J)** | - | - | - | - | - | - | - | - | - | - | - | - | - | - | - | - | 0.00 | 0.00 |
| **PC3 (J)** | - | - | - | - | - | - | - | - | - | - | - | - | - | - | - | - | - | 0.21 |
| **S3 (J)** | - | - | - | - | - | - | - | - | - | - | - | - | - | - | - | - | - | - |

The 16S rRNA gene sequence composition between libraries was compared by using the P-Test in the UniFrac. The P values shown in the table as 0.00 mean there is a significant difference between two compared libraries. Differences between two libraries which were not significant were labeled in red.
